# Trends in Racial and Ethnic Disparities in the Receipt of Lifesaving Procedures for Hospitalized Patients With Decompensated Cirrhosis in the US, 2009-2018

**DOI:** 10.1001/jamanetworkopen.2023.24539

**Published:** 2023-07-20

**Authors:** Lauren D. Nephew, Shannon M. Knapp, Kawthar A. Mohamed, Marwan Ghabril, Eric Orman, Kavish R. Patidar, Naga Chalasani, Archita P. Desai

**Affiliations:** 1 Division of Gastroenterology and Hepatology, Department of Medicine, Indiana University School of Medicine, Indianapolis; 2 Indiana University Simon Comprehensive Cancer Center, Indianapolis; 3 Division of Cardiovascular Medicine, Department of Medicine, Indiana University School of Medicine, Indianapolis; 4 Division of Medicine, University of Minnesota School of Medicine, Minneapolis; 5 Section of Gastroenterology, Department of Medicine, Baylor College of Medicine, Houston, Texas; 6 Michael E. DeBakey Veterans Affairs Medical Center, Houston, Texas

## Abstract

**Importance:**

Patients with decompensated cirrhosis are hospitalized for acute management with temporizing and lifesaving procedures. Published data to inform intervention development in this area are more than a decade old, and it is not clear whether there have been improvements in disparities in the receipt of these procedures over time.

**Objective:**

To evaluate the associations of race and ethnicity with receipt of procedures to treat decompensated cirrhosis over time in the US.

**Design, Setting, and Participants:**

This retrospective cross-sectional study analyzed National Inpatient Sample data on cirrhosis admissions among patients with portal hypertension–related complications from 2009 to 2018. All hospital discharges for individuals aged 18 years and older from 2009 to 2018 were assessed for inclusion. Admissions were included if they contained at least 1 cirrhosis-related *International Classification of Diseases, Ninth Revision, Clinical Modification (ICD-9-CM)* or *International Statistical Classification of Diseases, Tenth Revision, Clinical Modification (ICD-10-CM)* code and at least 1 cirrhosis-related complication *ICD-9-CM* or *ICD-10-CM* code (ie, ascites, hepatic encephalopathy, variceal hemorrhage [VH], and hepatorenal syndrome [HRS]). Data were analyzed from January to June 2022.

**Exposure:**

Hospitalization for decompensated cirrhosis.

**Main Outcomes and Measures:**

The outcomes of interest were trends in the odds ratios (ORs) for receiving procedures (upper endoscopy, transjugular portosystemic shunt [TIPS], hemodialysis, and liver transplantation [LT]) for decompensated cirrhosis and mortality by race and ethnicity, modeled over time. Multivariable logistic regression was used to assess these outcomes.

**Results:**

Among 717 580 admissions (median [IQR] age, 58 [52-67] years), 345 644 patients (9.8%) were Black, 623 991 patients (17.6%) were Hispanic, and 2 340 031 patients (47.4%) were White. Based on the modeled trends, by 2018, there were no significant differences by race or ethnicity in the odds of receiving upper endoscopy for VH. However, Black patients remained less likely than White patients to undergo TIPS for VH (OR, 0.54; 95% CI, 0.47-0.62) and ascites (OR, 0.34; 95% CI, 0.31-0.38). The disparity in receipt of LT improved for Black and Hispanic patients over the study period; however, by 2018, both groups remained less likely to undergo LT than their White counterparts (Black: OR, 0.66; 95% CI, 0.61-0.70; Hispanic: OR, 0.74; 95% CI, 0.70-0.78). The odds of death in Black and Hispanic patients declined over the study period but remained higher in Black patients than White patients in 2018 (OR, 1.08; 95% CI, 1.05-1.11).

**Conclusions and Relevance:**

In this cross-sectional study of individuals hospitalized with decompensated cirrhosis, there were racial and ethnic disparities in receipt of complex lifesaving procedures and in mortality that persisted over time.

## Key Points

**Question:**

What are the trends in receipt of procedures for hospitalized patients with decompensated cirrhosis by race and ethnicity in the United States?

**Findings:**

In this cross-sectional study 717 580 admissions for decompensated cirrhosis, there were no racial disparities in receipt of upper endoscopy for variceal hemorrhage by 2018; however, compared with White patients, Black patients remained less likely to receive transjugular portosystemic shunt for variceal hemorrhage and ascites. In 2018, both Black and Hispanic patients remained less likely to receive liver transplant, and Black patients had higher odds of death.

**Meaning:**

These findings suggest that racial and ethnic disparities in receipt of complex life-saving procedures and in mortality in the US persisted over time.

## Teaser

This cross-sectional study evaluates trends in receipt of procedures to treat decompensated cirrhosis by race and ethnicity from 2009 to 2018 in the US.

## Introduction

The complications of decompensated cirrhosis, including ascites, variceal hemorrhage (VH), and hepatic encephalopathy (HE), contribute to cirrhosis being the 12th leading cause of death in the US.^1^ These complications often require hospitalization for acute management with temporizing and lifesaving procedures. Obtaining access to care for chronic liver disease and liver transplant (LT) continues to be a challenge for minoritized racial and ethnic groups, including Black and Hispanic patients, in the modern era.^2,3^ In 2007, Nguyen et al^4^ described a cohort of hospitalized patients and recognized significant racial and ethnic disparities in the placement of transjugular intrahepatic portosystemic shunts (TIPS) and time to esophagogastroduodenoscopy (EGD) for VH from 1999 to 2003.^4^ To our knowledge, there has been no characterization of disparities in receipt of procedures for patients with cirrhosis since that time. However, there have been significant changes in cirrhosis care, including in the epidemiology, advances in treatment, efforts to improve the quality of care, and broad efforts to improve health care access with the expansion of Medicaid.^5,6,7,8^ It is imperative to understand what progress, if any, has been made toward improving disparities in the care of this high-risk population, so that public health and equity interventions can be appropriately targeted.

In this study, we aim to use a large nationwide sample to explore the associations of race and ethnicity with trends in receipt of procedures for decompensated cirrhosis, including TIPS, EGD, hemodialysis, and LT, as well as mortality.

## Methods

### Study Design

This cohort study was deemed exempt from human participants research review and informed consent by the Indiana University Human Research Protection Program, as defined in 45 CFR 46.102(f). Data were obtained from the Healthcare Cost and Utilization Project (HCUP) National Inpatient Sample database (NIS) between 2009 and 2018. The HCUP NIS has been extensively described elsewhere.^9,10,11,12,13^ This study is reported following the Strengthening the Reporting of Observational Studies in Epidemiology (STROBE) reporting guideline.

All hospital discharges for individuals aged 18 years and older from 2009 to 2018 were assessed for inclusion. Admissions were included if they contained at least 1 cirrhosis-related *International Classification of Diseases, Ninth Revision, Clinical Modification (ICD-9-CM)* and *International Statistical Classification of Diseases, Tenth Revision, Clinical Modification (ICD-10-CM)* codes and at least 1 cirrhosis-related complication *ICD-9-CM* or *ICD-10-CM* code (ie, ascites, HE, VH, and hepatorenal syndrome [HRS]).^9,10,11,12,14^ eFigure 1 in Supplement 1 provides the flowchart of study participants. As the study spans the transition in the NIS sampling method in 2012, we used adjusted trend weights as recommended by HCUP.^15^ The discharges were further categorized by race, ethnicity, and if procedures of interest (ie, EGD, TIPS, hemodialysis, and LT) were coded for during the hospitalization (eTable 1 in Supplement 1). We reviewed prior literature and medical coding references,^16,17^ as well as cross-referencing with clinical coding specialists to identify *ICD-9-CM* and *ICD-10-CM* codes when previously validated codes were not available.^18^

### Risk Factor and Outcome Variables

Our primary objective was to determine trends in the odds ratios (ORs) of receipt of procedures during the hospitalization, including EGD for VH, TIPS for VH and ascites, hemodialysis for acute kidney injury (AKI) or HRS, and LT. Secondary objectives were to examine the projected ORs for each procedure and mortality by race or ethnicity for 2018 and examine the annual standardized procedure rates (ASPRs) in 2018. Trends were assessed by racial or ethnic group: Black, Hispanic, White, and other (eg, American Indian or Alaska Native, Asian or Pacific Islander, and other not specified). These groups were defined using the race variable uniform definition in the NIS.^19^ Admission with missing or invalid data for the race variable were excluded from the analysis. Demographic data and *ICD-9-CM* and *ICD-10-CM* codes for relevant covariates were also extracted (eTable 1 in Supplement 1).^20,21^

### Statistical Analysis

The unit of analysis was each unique admission. Summary statistics for categorical variables were reported using proportions; χ^2^ tests were used to test for differences in the distribution of categorical variables across race. *P* values were 2-sided, and *P* < .05 was considered significant. Univariate associations of each categorical variable with outcomes were assessed using simple logistic regression. ASPRs were calculated by dividing the number of the procedures in a particular group by all the admissions with the condition requiring the procedure in the corresponding group multiplied by 1000. For example, the ASPR of EGD for VH in Black patients was calculated by dividing the number of EGDs for VH in Black patients, with all VH admissions in Black patients multiplied by 1000.

To assess trends in ORs by race and ethnicity over time, we used logistic regression. For mortality and LT outcomes, analyses used the full cohort. For EGD, we subset the cohort to only admissions with VH. For TIPS, we subset the cohort to those with VH or ascites. For hemodialysis, we subset the cohort to admissions with AKI or HRS. All models included race and ethnicity, year, race and ethnicity × year interaction effect, and a core set of covariates (ie, age; gender; insurance; Elixhauser Comorbidity Index; hospital region, teaching, and urban status; cirrhosis etiology and complications; sepsis; AKI; and receipt of mechanical ventilation) (eFigure 2 in Supplement 1). The model of EGD excluded VH while including transfusion of red blood cells, platelets, and fresh frozen plasma. The model for TIPS in VH excluded ascites, and the model for ascites excluded VH; both models included an indicator of emergent admission (ie, admission from the emergency department or transferred in from a different acute care hospital). As heart failure is a specific contraindication to TIPS and is known to vary by race, we also excluded discharges with heart failure in these 2 models.^22^ The model for hemodialysis excluded HRS and AKI. Finally, models for LT and mortality used the full core set of covariates. These models were also used to estimate the ORs in 2018 of each outcome for Black, Hispanic, and other race or ethnicity patients compared with White patients.

All of the analyses incorporated sampling weights provided by HCUP.^13^ Analyses were conducted using SAS statistical software version 9.4 (SAS Institute) between January and June 2022.

## Results

The clinical characteristics of 3 544 636 admissions with decompensated cirrhosis included in the study are shown in the Table categorized by race or ethnicity group. All characteristics were statistically significant among groups. Most of the cohort was aged 45 to 64 years, with an overall median (IQR) age of 58 (52-67) years. By race and ethnicity, 345 644 patients (9.8%) were Black, 623 991 patients (17.6%) were Hispanic, 2 340 031 patients (47.4%) were White, and 234 970 patients (6.6%) identified as other race or ethnicity. Hospitalized women were more likely to be Black. White patients were least likely to have Medicaid and most likely to have Medicare compared with other racial and ethnic groups. Black patients were the most likely to have Elixhauser Comorbidity Index greater than 6 (Table). Specific to liver disease, etiological characteristics of liver disease varied significantly by racial and ethnic group, with viral hepatitis more common in Black patients (hepatitis V and hepatitis B viruses) and hepatitis B virus having the highest prevalence in patients of other races and ethnicities (Table). Ascites was the most common decompensation in the cohort, with the highest rates seen in Black patients. Rates of VH were highest in Hispanic patients in the cohort (Table). There was significant variation in the distribution of admissions seen by region. Most patients of Hispanic ethnicity were admitted at hospitals in the West, most Black patients were admitted at hospitals in the South, and most patients of other races and ethnicities were admitted at hospitals in the West. Finally, White patients were more likely to be admitted to rural, nonteaching hospitals (Table).

**Table.** Characteristics of Admissions with Cirrhosis From 2009 to 2018 by Race and Ethnicity Group

Characteristic	Admissions, %^a^			
	Black (n = 345 644)	Hispanic (n = 623 991)	White (n = 2 340 031)	Other (n = 234 970)^b^
Age, y				
Median (IQR)	58 (52-64)	57 (50-66)	59 (52-67)	58 (50-67)
18-44	9.6	13.0	9.1	14.2
45-64	66.1	59.4	58.2	54.9
≥65	24.4	27.6	32.7	30.9
Gender^c^				
Men	59.7	64.7	61.1	60.7
Women	40.3	35.3	38.8	39.3
Elixhauser Index, median (IQR)				
0-3	31.2	41.4	39.1	40.8
4-6	52.2	47.3	48.7	48.1
>6	16.6	11.2	12.2	11.1
Admissions by payer type				
Medicaid	31.3	32.4	20.4	30.9
Medicare	43.8	37.9	46.1	37.6
Private or HMO	15.2	15.7	23.3	19.1
Other	9.7	14.0	10.2	12.4
Disease etiology^d^				
HCV	40.4	28.5	24.7	24.9
NASH	24.8	28.2	25.4	27.4
Alcohol-related liver disease	47.2	50.7	50.6	46.5
HBV	3.1	1.3	1.4	6.5
Cholestatic (PBC or PSC)	1.6	1.7	2.1	2.2
Autoimmune	1.9	1.5	1.2	1.5
Other	4.9	4.9	5.4	5.3
Decompensations				
Ascites	61.3	54.7	58.7	57.6
Hepatic Encephalopathy	39.3	41.2	42.1	40.3
Variceal bleeding	10.4	15.2	12.2	14.6
HCC	7.3	6.6	4.8	8.9
Hepatorenal syndrome	6.4	6.4	6.6	6.9
AKI	34.7	27.7	30.3	30.8
Sepsis	13.9	13.8	13.1	15.5
Mechanical ventilation	13.4	11.1	12.0	13.2
Hospital region				
Midwest	20.8	6.3	20.8	12.6
North East	19.2	13.3	18.4	20.6
South	49.0	36.3	41.4	26.9
West	11.0	44.2	19.5	39.8
Hospital status				
Rural	4.2	2.7	8.9	6.1
Teaching	74.5	65.3	59.6	67.7

Abbreviations: AKI, acute kidney injury; HBV, hepatitis B virus; HCC, hepatocellular carcinoma; HCV, hepatitis C virus; HMO, health maintenance organization; NASH, nonalcoholic steatohepatitis; PBC, primary biliary cholangitis; PSC, primary sclerosing cholangitis.

^a^ For all comparisons by groups, *P* < .001.

^b^ Other race or ethnicity includes Native American, Asian Pacific Islander, Other as defined by the race variable in Healthcare Cost and Utilization Project National Inpatient Sample data.

^c^ Rates of missing data for gender: 0.02% of Black patients, 0.01% of Hispanic patients, 0.01% of White patients, and 0.02% of patients identifying as other race or ethnicity.

^d^ Etiologies of liver disease are not mutually exclusive to allow admissions to be coded, for example, as HCV and alcoholic liver disease or NASH and alcoholic liver disease.

### Upper Endoscopy in Variceal Bleeding

**Figure 1.**
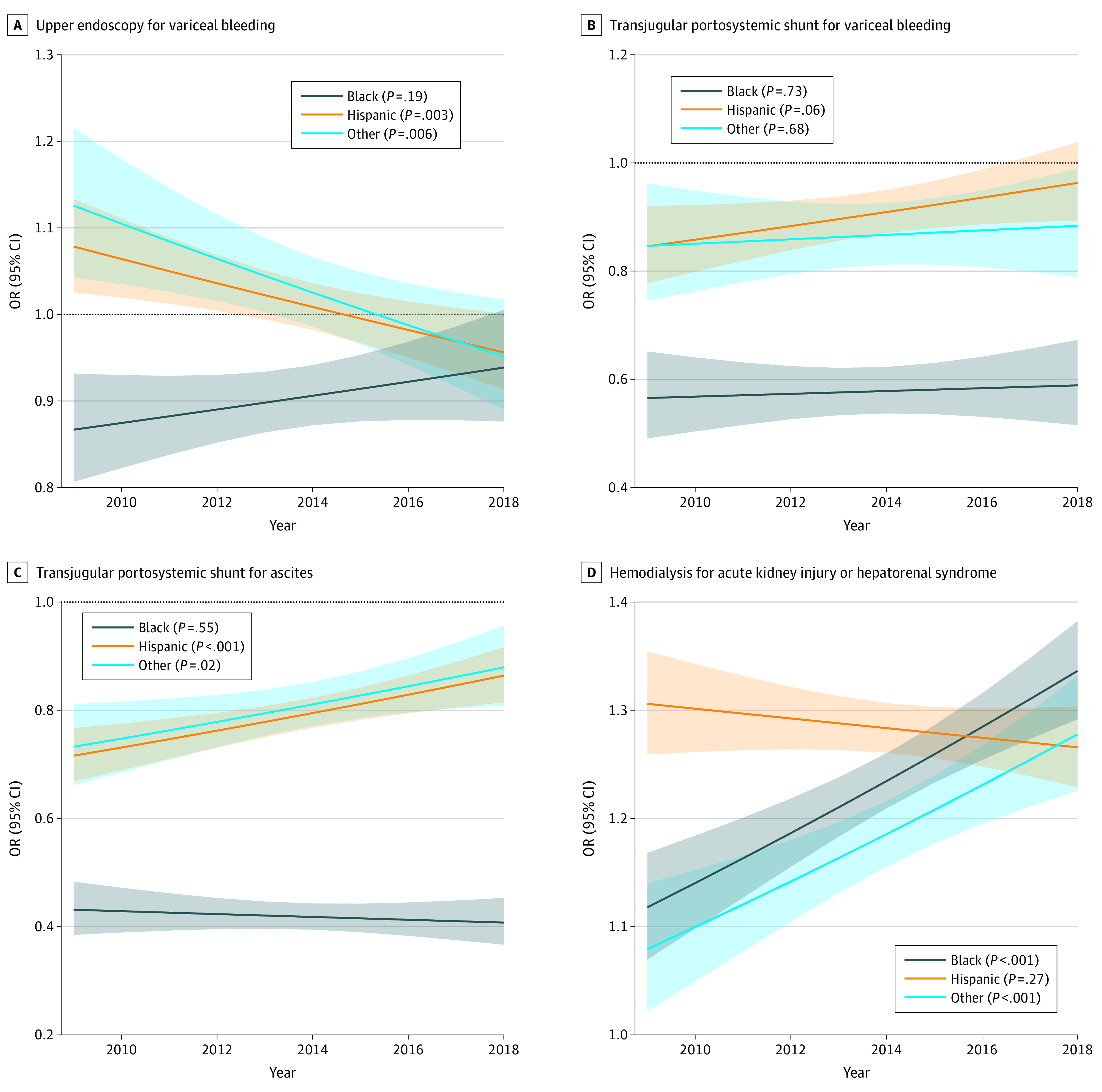
Trends in Treatments Over the Study Period for Racial and Ethnic Groups, by Treatment For all odds ratios (ORs), the reference group is White patients. Lines indicate estimates; shading, 95% CIs.

From 2009 to 2018 there was a significant decline in the odds of receiving upper endoscopy compared with White patients for VH in Hispanic patients (2009: OR, 1.08; 95% CI, 1.03-1.13; 2018: OR, 0.96; 95% CI, 0.91-1.00; *P* < .001) and patients of other races and ethnicities (2009: OR, 1.13; 95% CI, 1.04-1.22; 2018: OR, 0.95; 95% CI, 0.89-1.02; *P* < .001). In contrast, there was no significant change in the odds for receiving endoscopy for VH for Black patients (2009: OR, 0.87; 95% CI, 0.81-0.93; 2018: OR, 0.94; 95% CI, 0.88-1.01; *P* = .19) compared with White patients (Figure 1A; eTable 2 in Supplement 1). By 2018, the odds of receiving endoscopy for VH in Black, Hispanic, or patients from other racial and ethnic groups were not significantly different from White patients (eTable 2 in Supplement 1). In 2018, the ASPR of endoscopy for admissions with VH was 866.1 per 1000 admissions for Black patients, 879.1 per 1000 admissions for Hispanic patients, 859.4 per 1000 admissions for White patients, and 859.7 per 1000 admissions for patients of other races and ethnicities (eFigure 3 in Supplement 1).

### TIPS in VH and Ascites

From 2009 to 2018, there were no significant differences in the OR for receiving TIPS for VH for any racial or ethnic group compared with White patients (Figure 1B; eTable 2 in Supplement 1). By 2018, the odds of receiving TIPS for VH for Black patients were significantly lower than for White patients (OR, 0.57; 95% CI, 0.49-0.65). In 2018, the ASPR of TIPS in admissions with VH was 37.0 per 1000 admissions for Black patients, 46.6 per 1000 admissions for Hispanic patients, 58.0 per 1000 admissions for White patients, and 52.9 per 1000 admissions for patients of other races and ethnicities (eFigure 3 in Supplement 1).

The odds of receiving TIPS for ascites remained unchanged over the study period in Black patients compared with White patients (2009: OR, 0.43; 95% CI, 0.38-0.48; 2018: OR, 0.41; 95% CI, 0.37-0.45; *P* = .55) (Figure 1C; eTable 2 in Supplement 1). In contrast, the odds significantly increased for Hispanic patients compared with White patients (2009: OR, 0.72; 95% CI, 0.67-0.77; 2018: OR, 0.86; 95% CI, 0.81-0.96; *P* < .001) (Figure 1C; eTable 2 in Supplement 1). By 2018, there remained a significant disparity in the odds of receiving TIPS for ascites for Black patients (OR, 0.41; 95% CI, 0.37-0.45), Hispanic patients (OR, 0.86; 95% CI, 0.81-0.92), and patients of other race or ethnicity (OR, 0.88 95% CI, 0.81-0.96) compared with White patients. In 2018, the ASPR of TIPS in admissions with ascites was 7.2 per 1000 admissions for Black patients, 14.7 per 1000 admissions for Hispanic patients, 17.2 per 1000 admissions for White patients, and 14.2 per 1000 admissions for patients of other races and ethnicities (eFigure 3 in Supplement 1).

### Hemodialysis for AKI or HRS

The odds of receiving hemodialysis for AKI or HRS increased from 2009 to 2018 for Black patients (2009: OR, 1.12; 95% CI, 1.07-1.17; 2018: OR, 1.34; 95% CI, 1.29-1.38; *P* < .001) and patients of other races and ethnicities (2009: OR, 1.08; 95% CI, 1.02-1.14; 2018: OR, 1.28; 95% CI, 1.23-1.33; *P* < .001) compared with White patients (Figure 1D; eTable 2 in Supplement 1). By 2018, the odds of receiving hemodialysis for AKI or HRS was significantly higher for Black patients (OR, 1.34; 95% CI, 1.30-1.39), Hispanic patients (OR, 1.27; 95% CI, 1.23-1.30), and patients of other races and ethnicities (OR, 1.27; 95% CI, 1.23-1.30) compared with White patients. In 2018, the ASPR of hemodialysis for admissions with AKI or HRS was 118.9 per 1000 admissions for Black patients, 112.9 per 1000 admissions for Hispanic patients, 94.1 per 1000 admissions for White patients, and 116.9 per 1000 admissions for patients of other races and ethnicities (eFigure 3 in Supplement 1).

### LT for Hospitalized Patients With Decompensated Cirrhosis

**Figure 2.**
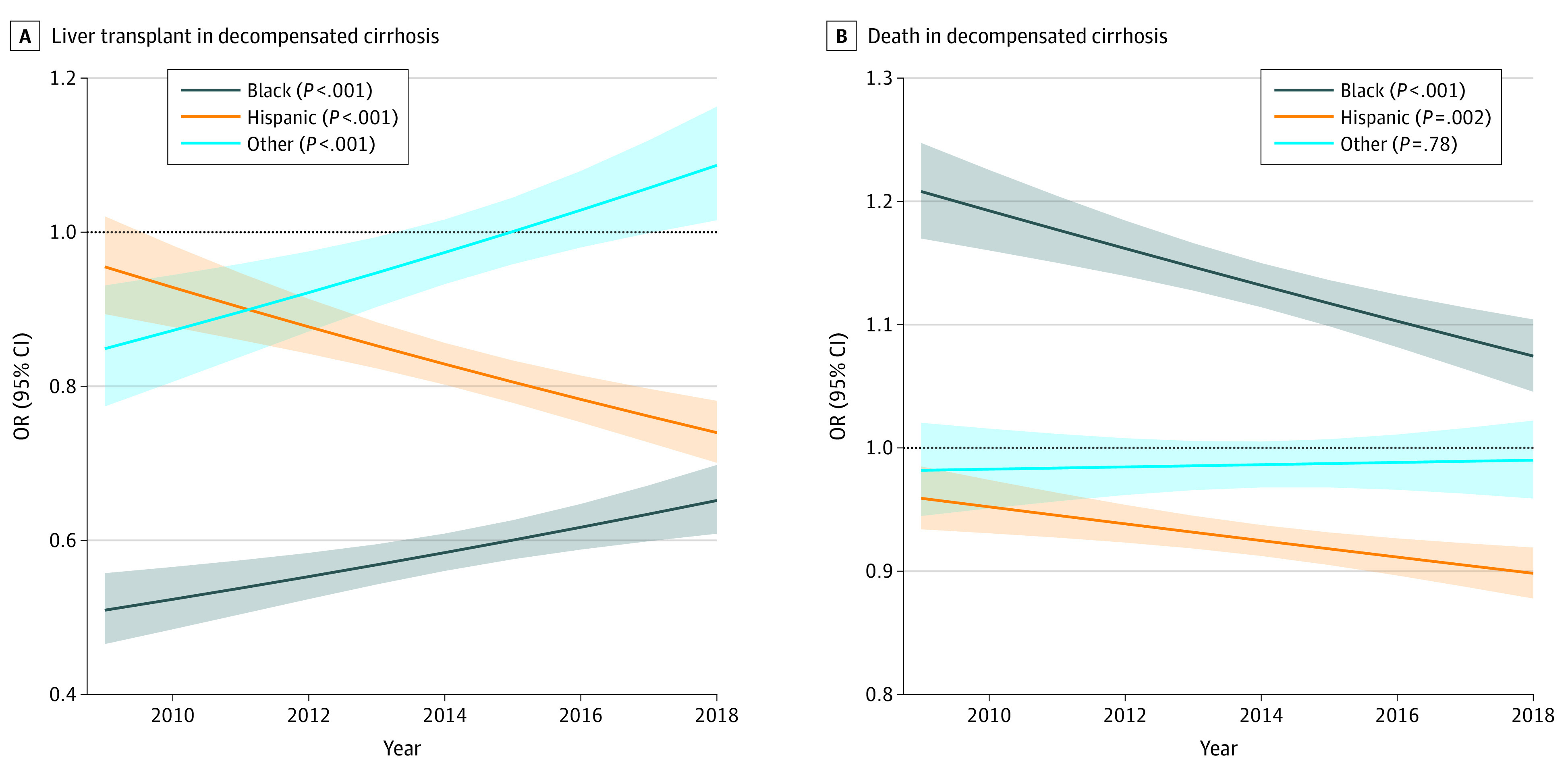
Trends in Odds of Liver Transplant or Death Over the Study Period for Racial and Ethnic Groups For all odds ratios (ORs), the reference group is White patients. Lines indicate estimates; shading, 95% CIs.

The odds of receiving LT decreased over the study period for Hispanic patients compared with White patients (2009: OR, 0.95; 95% CI, 0.89-1.02; 2018: OR, 0.74; 95% CI, 0.70-0.78; *P* < .001). In contrast, the odds of LT increased for Black patients and patients of other races and ethnicities compared with White patients (Figure 2A; eTable 2 in Supplement 1). By 2018, the OR for receiving LT remained significantly lower in Black patients (OR, 0.66; 95% CI, 0.61-0.70) and Hispanic patients (OR, 0.74; 95% CI, 0.70-0.78) compared with White patients. In 2018, the ASPR of LT for decompensated cirrhosis was 9.8 per 1000 admissions for Black patients, 9.6 per 1000 admissions for Hispanic patients, 11.6 per 1000 admissions for White patients, and 13.0 per 1000 admissions for patients of other races and ethnicities (eFigure 3 in Supplement 1).

### In-Hospital Mortality in Hospitalized Patients With Decompensated Cirrhosis

From 2009 to 2018 the odds of death in hospitalized patients with decompensated cirrhosis decreased significantly for Black patients (2009: OR, 1.21; 95% CI, 1.17-1.25; 2018: OR, 1.07; 95% CI, 1.05-1.10; *P* < .001) and Hispanic patients (2009: OR, 0.96; 95% CI, 0.93-0.98; 2018: OR, 0.90; 95% CI, 0.88-0.92; *P* = .002) compared with White patients (Figure 2B; eTable 2 in Supplement 1). By 2018, the odds of death remained significantly higher in hospitalized Black patients (OR, 1.08; 95% CI, 1.05-1.11), was significantly lower in Hispanic patients (OR, 0.90; 95% CI, 0.88-0.92), and was similar for patients of other races and ethnicities (OR, 0.99; 95% CI, 0.96-1.02) compared with White patients. In 2018, the in-hospital mortality rate in hospitalizations for decompensated cirrhosis was 88.3 per 1000 admissions for Black patients, 65.4 per 1000 admissions for Hispanic patients, 74.0 per 1000 admissions for White patients, and 84.9 per 1000 admissions for patients of other races and ethnicities (eFigure 3 in Supplement 1).

## Discussion

In this large contemporary and nationally representative cross-sectional study of hospitalized patients with cirrhosis, we observed important changes in trends in receipt of procedures and in mortality. By 2018, there were no significant differences in the odds of receiving upper endoscopy for VH among any racial and ethnic groups. In addition, patients in minoritized racial and ethnic groups had higher odds of receiving hemodialysis for AKI or HRS than White patients. However, when exploring more complicated, higher-risk procedures, disparities remained. Black and Hispanic patients had lower odds of receiving TIPS and LT than their White counterparts. Furthermore, while mortality decreased for Black patients, the 2018 odds of death remained higher than for White patients. Taken together, these findings suggest that there were disparities in receipt of the most complex but lifesaving procedures that persisted over the study period.

Black patients had lower odds of undergoing TIPS for VH than White patients. These differences persisted even after adjusting for comorbidity and history of hepatic encephalopathy and excluding patients with heart failure. Indications for emergent TIPS vary but most often include acute VH or persistent or failed endoscopic therapy of an acute VH.^23^ To our knowledge, there is no data to suggest Black patients have better endoscopic success rates in the setting of esophageal VH and therefore are less likely to need early or rescue TIPS. While we controlled for liver disease complications consistent with the Child-Turcotte-Pugh score as well as AKI, it is possible that there were disease severity or a comorbid condition variables that was not captured in NIS data that precluded Black patients from TIPS candidacy. However, if this is the case, it is a question that warrants further study. Other potential hypotheses, including lower procedure offer rates, access to interventional radiology consultation, and accessible education about the procedure, also warrant exploration. Understanding barriers to TIPS is important as we strive for health equity in the care of patients with cirrhosis through initiatives like the Cirrhosis Quality Collaborative (CQC).^24^

In this study, we found that in a cohort of patients who all had indications for LT and controlling for comorbidity and liver disease complications, there were a lower odds of receiving LT in 2018 for Black and Hispanic patients compared with White patients. Certainly, every patient with decompensated cirrhosis who is admitted to the hospital does not undergo LT, but a subset of the most ill will undergo expedited evaluation and LT.^25^ Reasons for disparities in the receipt of LT may include the social and structural determinants of health and the resultant sequalae of poor social support, high burden of disease, and substance use.^2,26,27^

While there were racial and ethnic disparities in receiving TIPS and LT, patients in minoritized racial and ethnic groups were more likely than White patients to receive hemodialysis for AKI or HRS. While there are potentially many hurdles to LT, including insurance approval, sustained social support, sobriety, and medical clearance, hemodialysis is covered by Medicaid and Medicare and has relatively fewer contradictions compared with LT.^28^ It is within expectations to see higher hemodialysis use in Black patients, given more baseline chronic kidney disease in this population; however, need does not always drive procedure receipt. Furthermore, given the disparity identified in LT, patients in minoritized racial and ethnic groups with decompensated cirrhosis may be more likely to be bridged with hemodialysis. Finally, there is data to suggest less palliative care use in patients in minoritized racial and ethnic groups, which may also contribute to higher hemodialysis use in patients with HRS who do not transition to hospice care.^29^

Notably, over the study period, the odds of death decreased for both Black and Hispanic patients. However, by 2018, Black patients had significantly higher odds of mortality than their White counterparts, while Hispanic patients had comparatively lower odds of mortality. Worse mortality among Black patients with cirrhosis was demonstrated in a 2021 metropolitan cohort of patients.^30^ In our cohort, Hispanic patients had an 11% lower risk of in-hospital mortality than White patients. Hispanic or Latinx American patients have been described in other data sets to have higher survival than their non-Hispanic White counterparts.^31^ Our findings warrant further study of the specific cultural, behavioral, social, and biologic determinants of this observation.^31^

### Next Steps

NIS data provide up-to-date information to guide the next steps in this analysis seeking to understand and intervene on disparities in the receipt of therapies for end-stage liver disease. Using a socioecological model as a framework to target action “from upstream to downstream,”^32^ we propose some next steps to understand and address these disparities. First, antiracism and unconscious bias training should be undertaken for all physicians and health care staff, followed by an evaluation of discussion rates to ensure patients of all races and ethnicities are being offered an opportunity to explore these procedures. Second, advocacy efforts from our national societies and from individual physicians to expand Medicaid coverage in nonexpansion states so that subspecialty care is more accessible to all. Third, prospective trials are needed that include safety-net and community hospitals and explore the impacts of the social determinants of health and social needs on being able to reach tertiary care centers that provide treatments. These interventions should be informed by minoritized communities, as qualitative and mixed method studies that allow patients to be a part of the solution are critical. Fifth, the evidence is clear that multilevel interventions have been the most successful at improving health care disparities, so solutions will need to consider intervening on both upstream and downstream factors to be effective.^33^ Finally, the most important next is to acknowledge that disparities will not resolve without intentional action.

### Limitations

While there are important strengths to our analysis using a decade-long, nationally representative data set, we acknowledge several limitations, particularly in relation to the use of an administrative data set. While discharge codes have been validated to identify groups of admission with cirrhosis and liver-related procedures, our study does span the transition from *ICD-9* to *ICD-10* coding. All codes were carefully cross-mapped between *ICD-9-CM* and *ICD-10-CM* by us as well as by professional coders, but the transition to *ICD-10* may introduce variations in coding.^34^ Moreover, the NIS lacks laboratory data, such as Model for End-Stage Liver Disease score, an important marker of severity of liver disease. Nonetheless, we were able to include complications key to the Child-Turcotte-Pugh score, as well as comorbidities and AKI or HRS, which account largely for differences in disease severity and should not explain disparities as large as 30% to 40%. Furthermore, we were not able to evaluate social support, substance use history, and other nonclinical determinants of access to complex procedures, like LT. These are variables that contribute to and confound the evaluation of racial disparities in LT and will need to be explored to inform interventions. As the data were deidentified, we were unable to account for rehospitalizations and subsequent receipt of procedures during follow up outpatient care. Therefore, it is possible a particular racial or ethnic group is more likely to receive a TIPS or EGD as outpatient vs during the inpatient encounter; however, these should not diminish the racial and ethnic differences noted in this study, given our evaluation of conditions that warrant urgent consideration of therapeutic procedures. These limitations are inherent to large national data sets, such as the NIS, and offset the strengths of the study.

## Conclusions

This cohort study found that, while there were no disparities in receipt of upper endoscopy for VH, there were persistent racial and ethnic disparities in receipt of more complicated inpatient procedures. While these procedures are more complex and higher risk, they are also lifesaving for patients with decompensated cirrhosis. Targeted efforts will be required to ensure that another decade does not pass without improved equity in these lifesaving procedures.

## Notes

Supplement 1. **eTable 1.** ICD-9 and ICD-10 Codes Used for Inclusion, Exclusion and Procedure Identification **eTable 2.** Trend in Adjusted Odds Ratios for Procedures of Interest by Racial and Ethnic Group as Depicted in Figures 1 and 2 **eFigure 1.** Study Participation Flow Diagram **eFigure 2.** Covariates Included in Model to Calculate Adjusted Odds Ratio for Each Procedure **eFigure 3.** Annual Standardized Procedures and Mortality Rates by Race-Year

Supplement 2.
Data Sharing Statement
