# Error in Key Points and Abstract

**DOI:** 10.1001/jamanetworkopen.2023.31115

**Published:** 2023-08-16

In the Original Investigation titled “Trends in Racial and Ethnic Disparities in the Receipt of Lifesaving Procedures for Hospitalized Patients With Decompensated Cirrhosis in the US, 2009-2018,”^1^ published July 20, 2023, there was an error in the sample size described in the Key Points and Abstract. The correct sample size is 3 544 636 admissions. This article has been corrected.^1^
