# Supplementary material for: Trends in Racial and Ethnic Disparities in the Receipt of Lifesaving Procedures for Hospitalized Patients With Decompensated Cirrhosis in the US, 2009-2018
Source: JAMA Netw Open. 2023 Jul 20;6(7):e2324539. doi: 10.1001/jamanetworkopen.2023.24539 (PMC10359964; doi:10.1001/jamanetworkopen.2023.24539)
Supplement: Supplement 1. — eTable 1. ICD-9 and ICD-10 Codes Used for Inclusion, Exclusion and Procedure Identification eTable 2. Trend in Adjusted Odds Ratios for Procedures of Interest by Racial and Ethnic Group as Depicted in Figures 1 and 2 eFigure 1. Study Participation Flow Diagram eFigure 2. Covariates Included in Model to Calculate Adjusted Odds Ratio for Each Procedure eFigure 3. Annual Standardized Procedures and Mortality Rates by Race-Year [file jamanetwopen-e2324539-s001.pdf]

Supplemental Online Content

Nephew LD, Knapp SM, Mohamed KA, et al. Trends in racial and ethnic disparities in the receipt of lifesaving procedures for hospitalized patients with decompensated cirrhosis in the US, 2009-2018. *JAMA Netw Open*. 2023;6(7):e2324539. doi:10.1001/jamanetworkopen.2023.24539

eTable 1. ICD-9 and ICD-10 Codes Used for Inclusion, Exclusion and Procedure Identification
eTable 2. Trend in Adjusted Odds Ratios for Procedures of Interest by Racial and Ethnic Group as Depicted in Figures 1 and 2
eFigure 1. Study Participation Flow Diagram
eFigure 2. Covariates Included in Model to Calculate Adjusted Odds Ratio for Each Procedure
eFigure 3. Annual Standardized Procedures and Mortality Rates by Race-Year

This supplemental material has been provided by the authors to give readers additional information about their work.

© 2023 Nephew LD et al. *JAMA Network Open.*

eTable 1. ICD9 and 10 Codes used for inclusion, exclusion and procedure identification

Condition	ICD9 Codes	ICD10 codes
**Cirrhosis**	571.2, 571.5, 571.6	K74*, K70.3*, K70.2
**Complications**		
Ascites	789.5* but not 789.51	R18.8, K70.31, K70.11, K71.51
Variceal hemorrhage	456.0, 456.20	I85.01, I 85.11
Hepatic encephalopathy	348.31, 348.30, 348.39, 572.2, 070.2, 070.4, 070.6, 070.71, 780.09	G93.40, G93.41, G93.49, R40, K70.41, K71.11, K72.01, K72.11, K72.91, B19.0, B19.11, B19.21
Hepatorenal Syndrome	572.4	K76.7
HCC	155.0	C22.0, C22.8, C229
Hepatorenal syndrome	572.4	K76.7
**Procedures**		
Upper Gastrointestinal Endoscopy	45.13, 45.14, 45.16, 45.30, 45.34, 42.23, 42.24, 42.92, 43.41, 44.13, 44.14, 44.22, 44.43, 46.85, 49.95, 99.29, 98.02, 98.03	0DJ08ZZ, 0D983ZX, 0D958ZX, 0DJ08ZZ, 0D953ZX, 0DJ68ZZ, 0D963ZX, 0DJ07ZZ, 0D568ZZ, 0D578ZZ, 0D588ZZ, 0D598ZZ 0D5A8ZZ, 0D718ZZ, 0D728ZZ, 0D738ZZ, 0D748ZZ, 0D758ZZ, 0D768ZZ, 0D778ZZ, 0D788ZZ, 0D798ZZ, 0D7A8ZZ, 0D718DZ, 0D728DZ, 0D738DZ, 0D748DZ, 0D758DZ, 0D768DZ, 0D778DZ, 0D788DZ, 0D798DZ, 0D7A8DZ, 0D9180Z, 0D918ZX, 0D918ZZ 0D9280Z, 0D928ZX, 0D928ZZ, 0D9380Z 0D938ZX, 0D938ZZ, 0D9480Z, 0D948ZX 0D948ZZ, 0D9580Z, 0D958ZX, 0D958ZZ 0D9680Z, 0D968ZX, 0D968ZZ, 0D9780Z 0D978ZX, 0D978ZZ, 0D9880Z, 0D988ZX 0D988ZZ, 0D9980Z, 0D998ZX, 0D998ZZ, 0D9A80Z, 0D9A8ZX, 0D9A8ZZ, 0DB18ZX, 0DB18ZZ, 0DB28ZX, 0DB28ZZ 0DB38ZX, 0DB38ZZ, 0DB48ZX, 0DB48ZZ 0DB58ZX, 0DB58ZZ, 0DB68ZX, 0DB68ZZ 0DB78ZX, 0DB78ZZ, 0DB88ZX, 0DB88ZZ 0DB98ZX, 0DB98ZZ, 0DBA8ZX, 0DBA8ZZ, 0DC18ZZ, 0DC28ZZ, 0DC38ZZ 0DC48ZZ, 0DC58ZZ, 0DC68ZZ, 0DC78ZZ 0DC88ZZ, 0DC98ZZ, 0DCA8ZZ, 0DJ08ZZ
Variceal Ligation or Other Control of bleeding	42.91, 42.33, 44.43, 49.95, CCS[#] Procedure Category 68	06L30ZZ, 0D554ZZ, 0D518ZZ, 0D528ZZ, 0D538ZZ, 0D548ZZ, 0D558ZZ, 0D568ZZ, 0W3P8ZZ, 3E0G8TZ, 0DQ78ZZ, 0DQ68ZZ, 0DQ28ZZ, 0DQ38ZZ, 0DQ58ZZ, 0DQ48ZZ, 06L38CZ, 06L34CZ 06L38ZZ, 3E0G8GC, 06L28CZ, 06L28ZZ

© 2023 Nephew LD et al. *JAMA Network Open.*

Condition	ICD9 Codes	ICD10 codes
Insertion of Sengstaken tube /esophageal tamponade	96.06	0DL57DZ, 0DL58DZ
Transjugular Intrahepatic Portosystemic shunt	39.1	06183J4, 06184J4, 06183JY, 061847Y<br>061849Y, 06184AY, 06184JY, 06184KY<br>06184ZY, 06183DY, 06184DY
Hemodialysis	39.95, V45.1, V45.11, V56.0, CCS# Procedure Category 58	Z992, Z4901, Z4931, 5A1D*
Liver Transplant	50.5*, 00.91, 00.92, 00.93	0FY00Z0, 0FY00Z1, 0FY00Z2
Red Blood Cell Transfusion	99.04	30233N1, 30243N1, 30233N0, 30233P0, 30233P1
Platelets Transfusion	99.05	30233R1, 30243R1, 30230R0
Fresh Frozen Plasma Transfusion	99.07	30233K1, 30243K1, 30233K0, 30233L0, 30233L1
**Etiology of Liver Disease**		
Alcohol	571.0, 571.1, 571.2, 571.3	K70*
NASH	571.5, 571.40, 571.41, 571.49<br>AND<br>Code for Diabetes% OR Obesity (V85.3* V85.4*, 278.00*, 278.01*, 278.02*)	K73.x, K74.60, K74.69<br>AND<br>Code for Diabetes% OR Obesity (Z68.3* Z68.4*, E66.0* E66.01* E66.09* E66.1* E66.2* E66.3* E66.8* E66.9*)
Hepatitis C	070.44, 070.54, 070.70, 070.71, V026.2	B18.2, B19.20, B19.21, Z22.52
Other	273.4, 275.1, 275.01, 275.03, 571.42, 571.6, 571.6, 571.6, 576.1, 070.49, 070.59, 07.06, 07.09, 573.3, 571.40, 571.41, 571.49, 571.49, 571.9, 573.9	E88.01, E83.00, E83.01, E83.09, E83.110, E83.118, K75.4, K74.3, K74.4, K74.5, K83.01, K83.09, B17.2, B17.8, B19.0, B19.9, K71.6, K73.9, K73.0, K73.2, K73.8, K74.1, K76.89, K76.9
HBV	070.20, 070.21, 070.22, 070.23, 070.32, 070.33, V02.61	B18.0, B18.1, Z22.51, B19.10, B19.11
Cholestatic	571.6, 576.1	K74.3, K74.4, K74.5, K83.01, K83.09, K83.8, K83.9
Autoimmune	571.42	K75.4
Other	273.4, 275.1, 275.01, 275.03, 070.49, 070.59, 070.6, 070.9, 573.3, 571.40, 571.41, 571.49 571.5, 571.8<br>571.9<br>573.0, 573.8, 573.9, 751.62, 453.0, 570, V02.60, V02.69, 070.43, 070.53	E88.01, E83.00, E83.01, E83.09, E83.110, E83.118, B17.2, B17.8, B17.9, B18.8, B18.9, B19.0, B19.9, K71*, K72*, K73*, K74.60, K74.69, K73.9, K73.0, K73.2, K73.8, K74.1, K75.3, K75.89, K75.9, K76.1, K76.3, K76.4, K76.5, K76.89, K76.9 K77*, Q44.6, I82.0, Z22.50, Z22.59
**Related conditions**		
Sepsis	CCS# category 2	A021, A207, A227, A267, A327, A392, A393, A394, A400, A401, A403, A408, A409, A4101, A4102,A411

© 2023 Nephew LD et al. *JAMA Network Open.*

Condition	ICD9 Codes	ICD10 codes
		A412, A413, A414, A4150, A4151, A4152,A4153, A4159 A4181, A4189, A419, A427, A5486, B007, B377, I76, O0337, O0387, O0487, O0737 O0882, O85, O8604, P360, P3610, P3619, P362, P3630 P3639, P364, P365, P368, P369, R6520, R6521, T8144XA T8144XD
AKI	584.5, 584.6, 584.7, 584.8, 584.9, 586, 572.4	K76.7* N17*
Mechanical Ventilation	528.81, 96.71, 96.70, 96.72, 96.04, CCS# procedure category 216	J96.00, J96.90, 09HN7BZ, 09HN8BZ 0BH13EZ, 0BH17EZ, 0BH18EZ, 0CHY7BZ, 0CHY8BZ,0DH57BZ, 0DH58BZ, 0WHQ73Z, 0WHQ7YZ 5A09357, 5A09457,5A09557 5A1935Z, 5A1945Z,5A1955Z
Heart Failure	398.91, 402.11, 402.91, 404.01, 404.11, 404.03, 404.13, 404.91, 404.93, 428x	I09.81, I11.0, I13.0, I13.2, I50x

***** indicates any codes starting with ICD code stem were include

% Diabetes diagnosis defined using Elixhauser indicators for diabetes or diabetes complications

Clinical Categorization software (CCS, https://www.hcup-us.ahrq.gov/toolssoftware/ccs/ccs.jsp)

© 2023 Nephew LD et al. *JAMA Network Open.*

eTable 2. Trend in adjusted odds ratios for procedures of interest by racial and ethnic group as depicted in Figure 1 and 2.

	**2009 Adjusted* Odds Ratio (95% CI)**	**2018 Adjusted* Odds Ratio (95% CI)**	**p-value for the trend over study period[&]**	**Disparity Changing Over Time?^**	**Disparity Present in 2018?[%]**
EGD in Variceal Bleeding[$]					
Black vs. White	0.87 (0.81, 0.93)	0.94 (0.88, 1.01)	0.19	–	✔
Hispanic vs. White	1.08 (1.03, 1.13)	0.96 (0.91, 1.00)	0.003	✓	✔
Other vs. White	1.13 (1.04, 1.22)	0.95 (0.89, 1.02)	0.006	✓	✔
TIPS for Variceal Bleeding[$#]					
Black vs. White	0.57 (0.49, 0.65)	0.59 (0.52, 0.67)	0.73	–	⚠
Hispanic vs. White	0.85 (0.78, 0.92)	0.96 (0.89, 1.04)	0.06	–	✔
Other vs. White	0.85 (0.74, 0.96)	0.88 (0.79, 0.99)	0.68	–	✔
TIPS for Ascites[@#]					
Black vs. White	0.43 (0.38, 0.48)	0.41 (0.37, 0.45)	0.55	–	⚠
Hispanic vs. White	0.72 (0.67, 0.77)	0.86 (0.81, 0.92)	< 0.001	✓	⚠
Other vs. White	0.73 (0.66, 0.81)	0.88 (0.81, 0.96)	0.02	✓	⚠
Hemodialysis for AKI/HRS[!]					
Black vs. White	1.12 (1.07, 1.17)	1.34 (1.29, 1.38)	<0.001	✗	⚠
Hispanic vs. White	1.31 (1.26, 1.35)	1.27 (1.23, 1.30)	0.27	–	⚠
Other vs. White	1.08 (1.02, 1.14)	1.28 (1.23, 1.33)	<0.001	✗	⚠
Liver Transplant					
Black vs. White	0.51 (0.47, 0.56)	0.65 (0.61, 0.70)	< 0.001	✓	⚠
Hispanic vs. White	0.95 (0.89, 1.02)	0.74 (0.70, 0.78)	<0.001	✗	⚠
Other vs. White	0.85 (0.77, 0.93)	1.09 (1.02, 1.16)	< 0.001	✓	✔
Death					
Black vs. White	1.21 (1.17, 1.25)	1.07 (1.05, 1.10)	<0.001	✓	⚠
Hispanic vs. White	0.96 (0.93, 0.98)	0.90 (0.88, 0.92)	0.002	✗	✔
Other vs. White	0.98 (0.94, 1.02)	0.99 (0.96, 1.02)	0.78	–	✔

* The OR are predicted values from the models presented in Figures 1 and 2 and are provides as examples to improve interpretation. All models included a core set of co-variates: race (Black, Hispanic, Other and White as the reference group); year; race-year interaction effect; age (18-44, 45-64, ≥65); gender; insurance (Medicaid, Medicare, Private, Other); modified ECI (0-3, 4-6, ≥7); hospital region (Midwest, Northeast, South, West); urban or rural hospital; teaching or non-teaching hospital; indicators of liver disease etiologies: HCV, HBV, NASH, Cholestatic, Alcohol, Other; indicators for cirrhosis complications: Ascites, VH, HCC, HRS, Sepsis, AKI; and Mechanical Ventilation.

& The overall trend was assessed over the study period and a $p < 0.05$ for this trend indicates that there was a significant change over time.

^ "✓" indicates disparity significantly narrowing over study period, "✗" indicates disparity significantly wider over study, "–" indicates disparity no different over study period

% ✔ indicates equivalent or better outcome compared to White individuals in 2018, ⚠ indicates worse outcomes compared to White individuals in 2018

$ model did not include indicator for VH and excluded those with HF (n=3,897)

model includes additional covariable indicator of whether the admission was emergent

@ model did not include indicator for ascites and excluded those with heart failure (n=69,787)

! model did not include indicator for AKI/HRS

© 2023 Nephew LD et al. *JAMA Network Open.*

eFigure 1. Study participation flow diagram

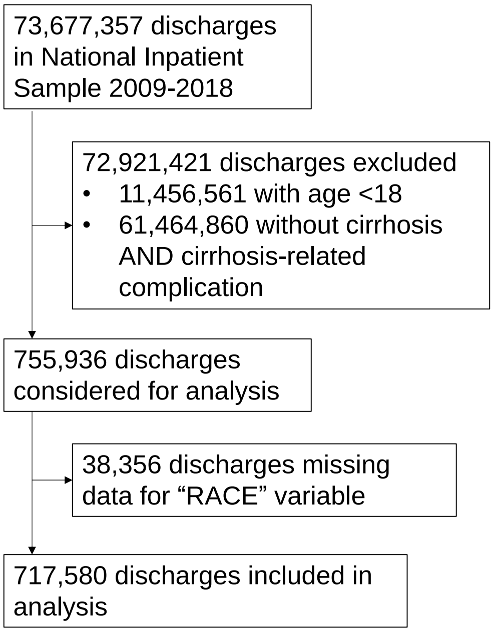

© 2023 Nephew LD et al. *JAMA Network Open.*

eFigure 2. Co-variates included in model to calculate adjusted odds ratio for each procedure (A. EGD for variceal bleeding, B. TIPS for variceal bleeding, C. TIPS for ascites, D. HD for AKI/HRS, E.LT for decompensated cirrhosis, F. Mortality in decompensated).

A. EGD for variceal bleeding

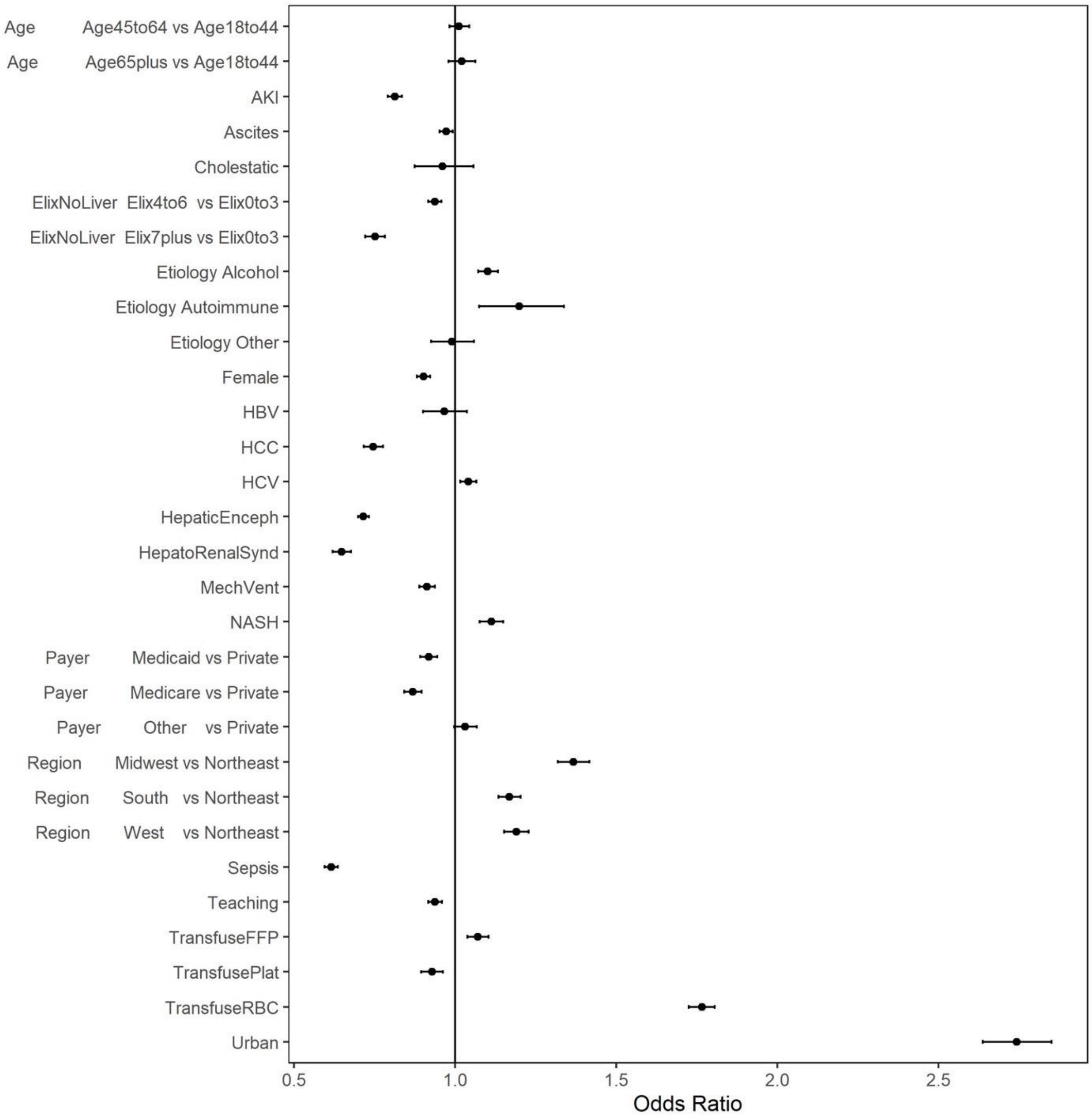

© 2023 Nephew LD et al. *JAMA Network Open.*

B. TIPS for variceal bleeding

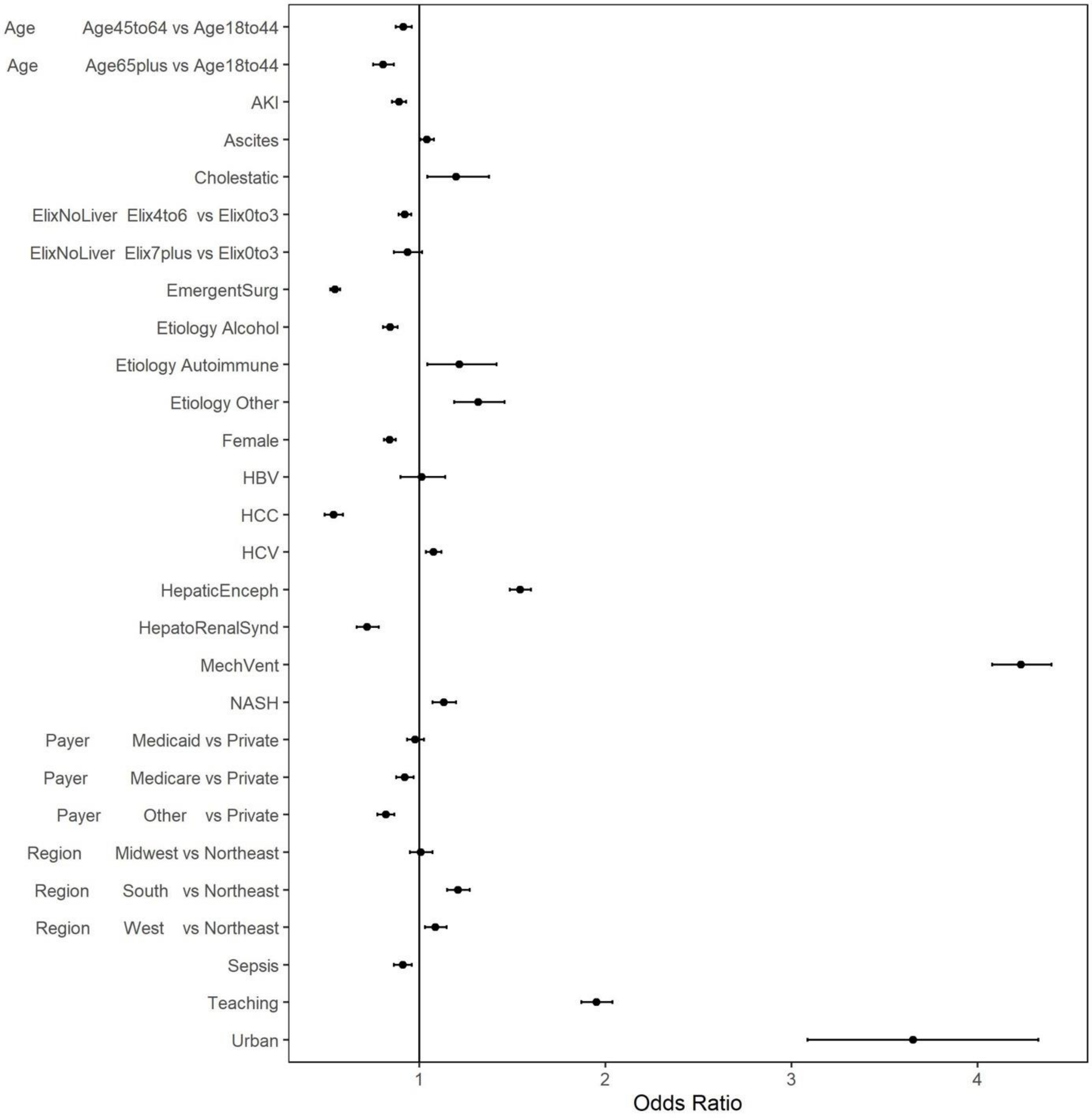

© 2023 Nephew LD et al. *JAMA Network Open.*

C. TIPS for ascites

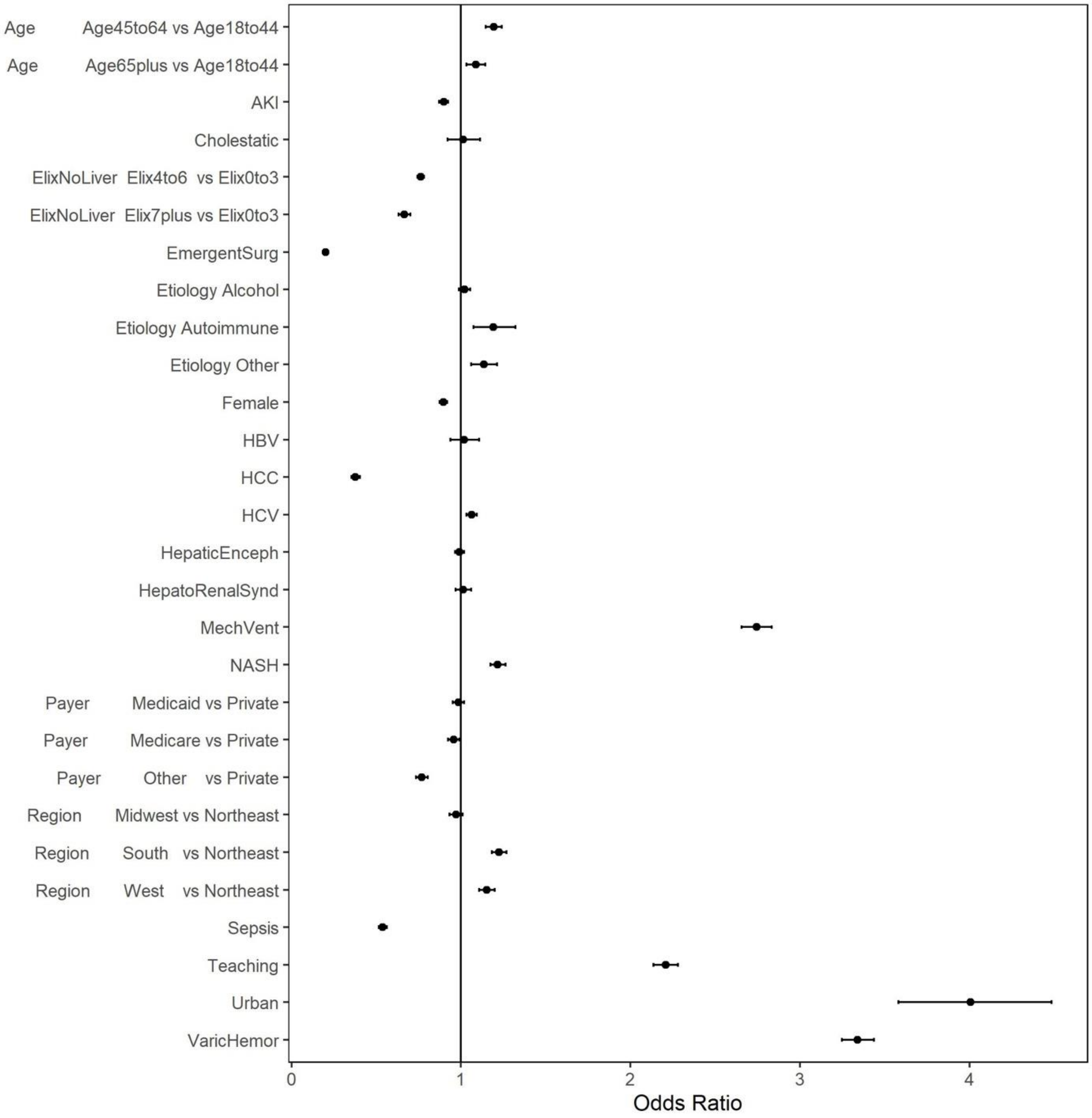

© 2023 Nephew LD et al. *JAMA Network Open.*

D. HD for AKI

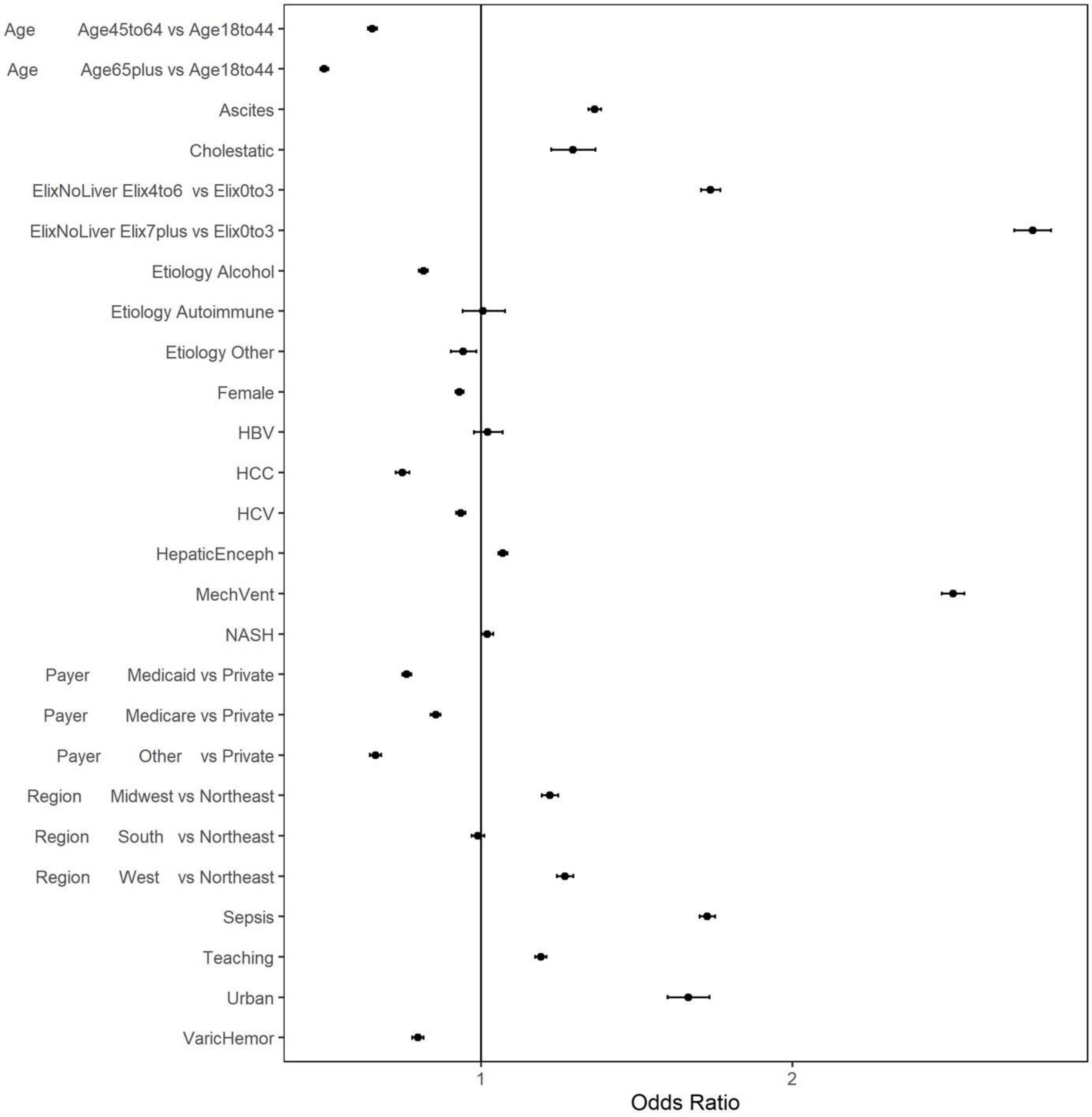

© 2023 Nephew LD et al. *JAMA Network Open.*

E. Liver Transplant (x-axis on logarithmic scale)

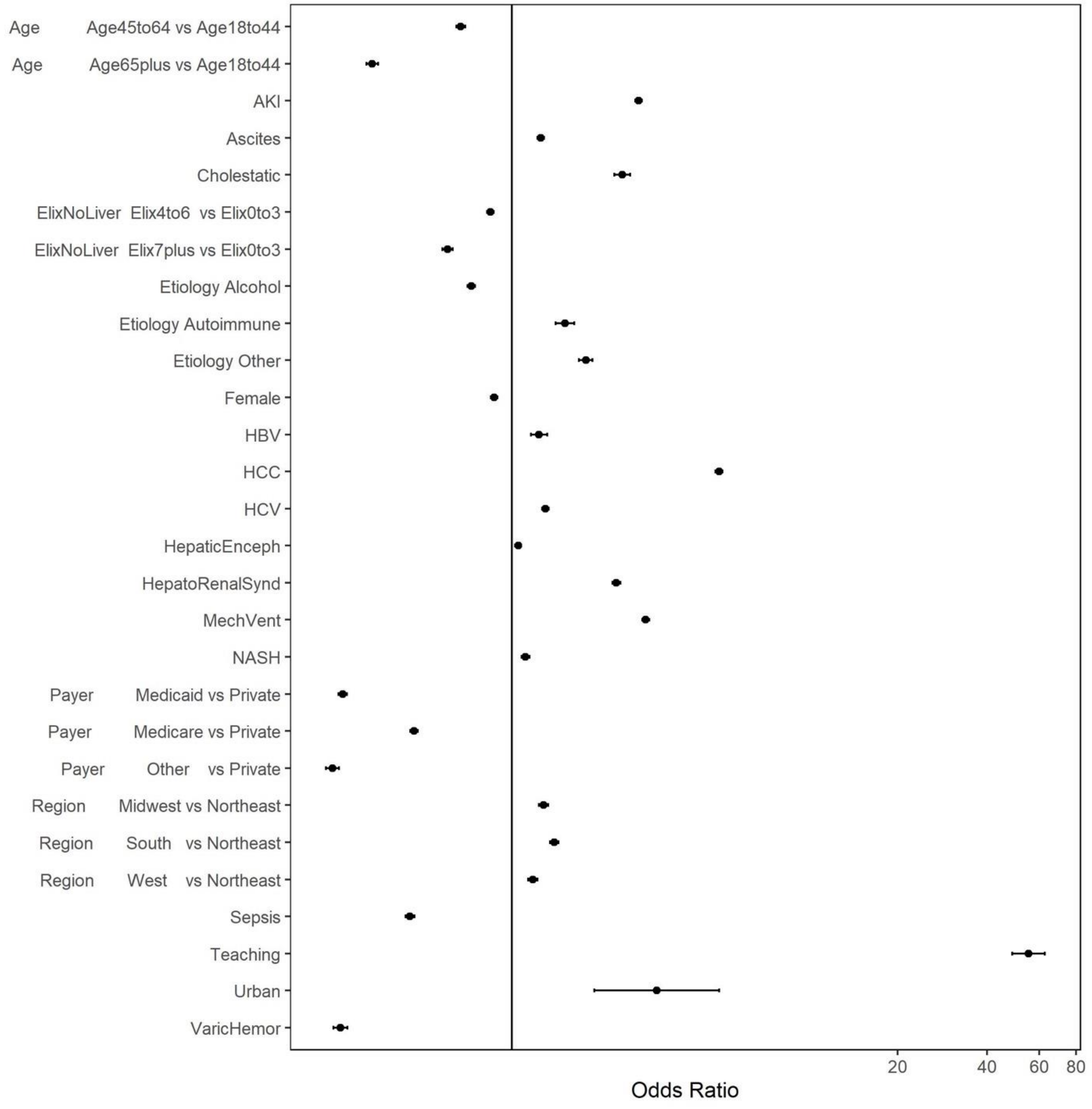

© 2023 Nephew LD et al. *JAMA Network Open.*

F. Mortality (x-axis on logarithmic scale)

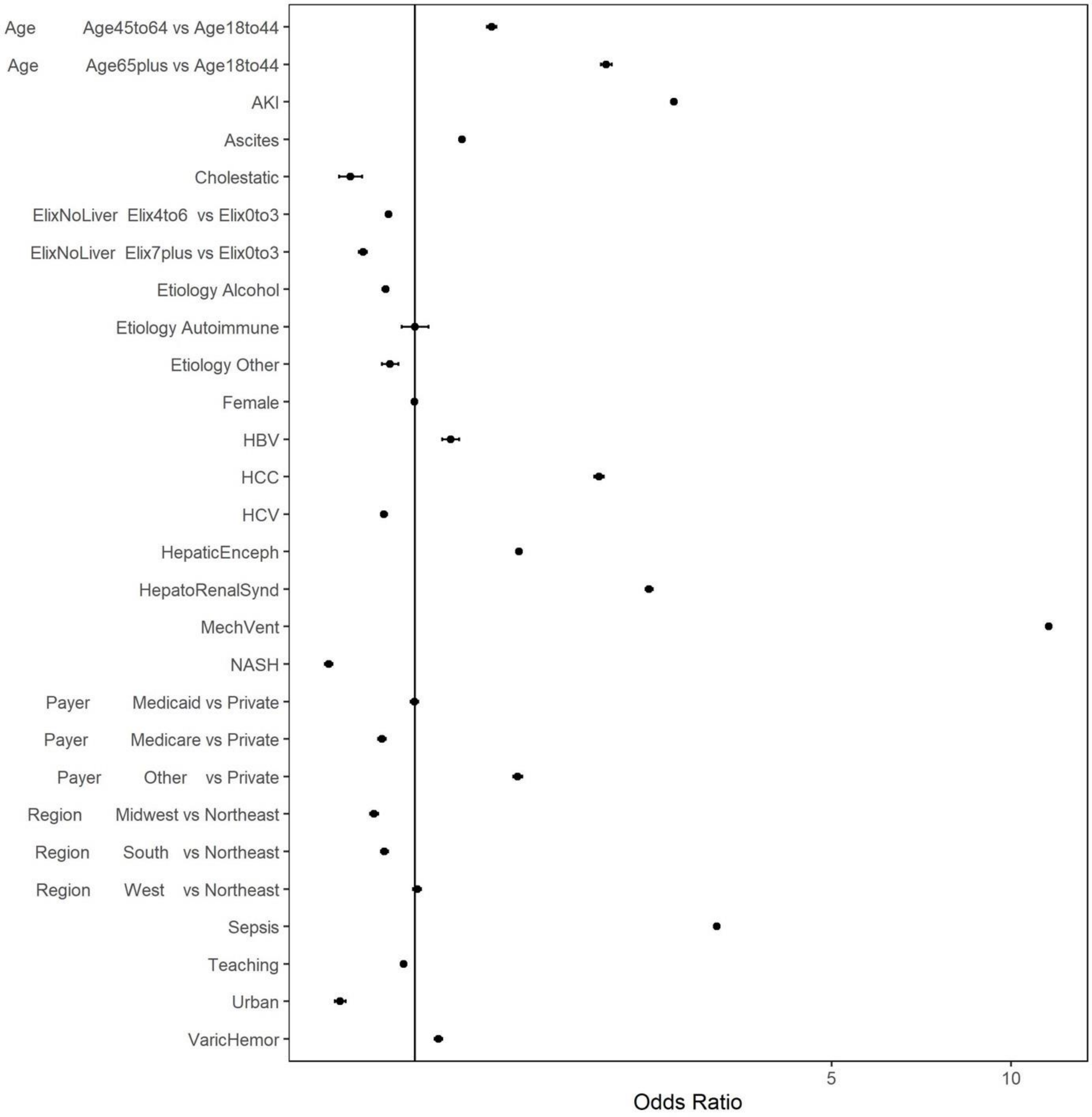

© 2023 Nephew LD et al. *JAMA Network Open.*

eFigure 3. Annual standardized procedures and mortality rates by race-year
(A. EGD for variceal bleeding, B. TIPS for variceal bleeding, C. TIPS for ascites, D. HD for AKI/HRS, E.LT for decompensated cirrhosis, F. Mortality in decompensated).

A. EGD for variceal bleeding

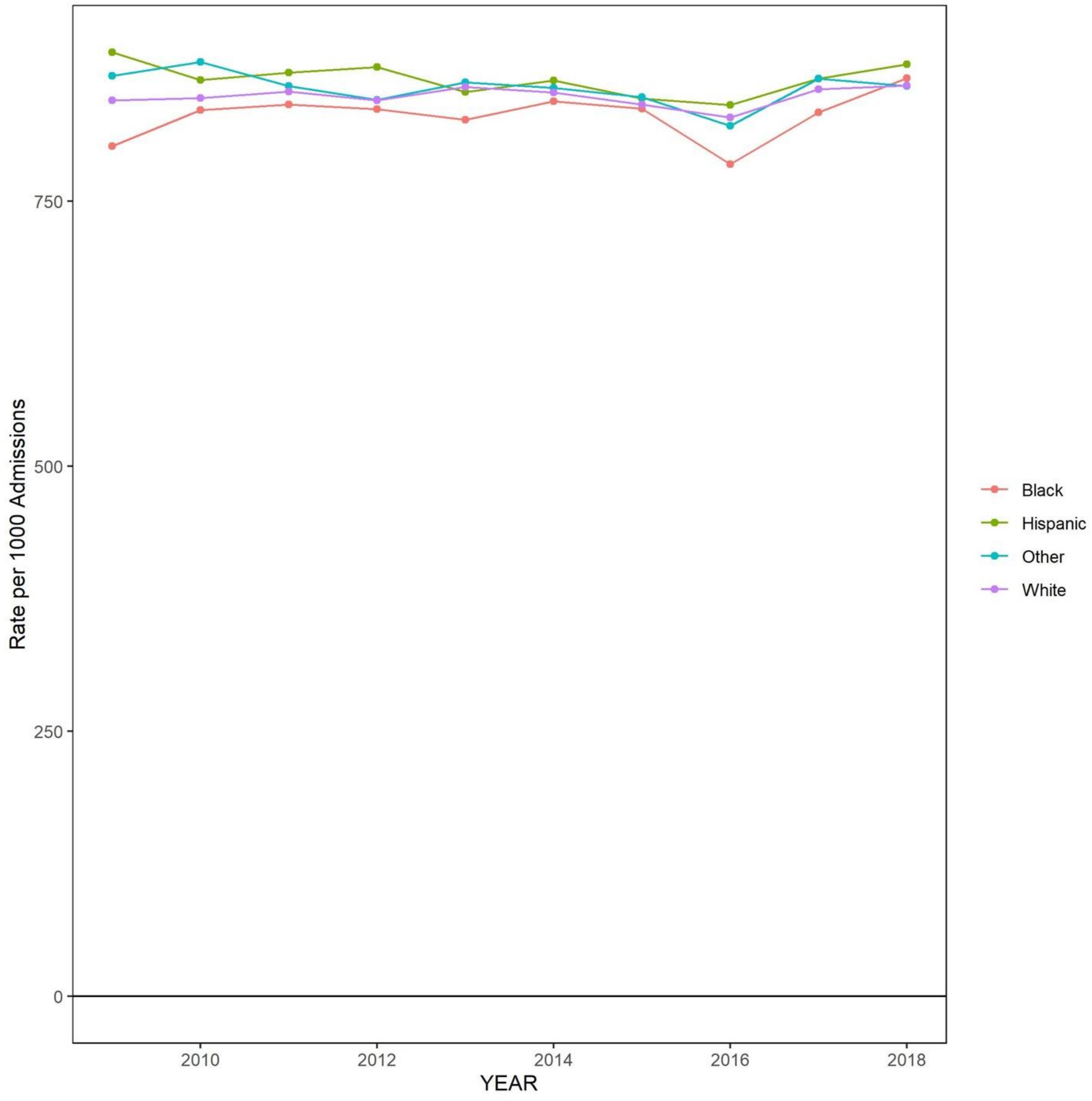

© 2023 Nephew LD et al. *JAMA Network Open.*

B. TIPS for variceal bleeding

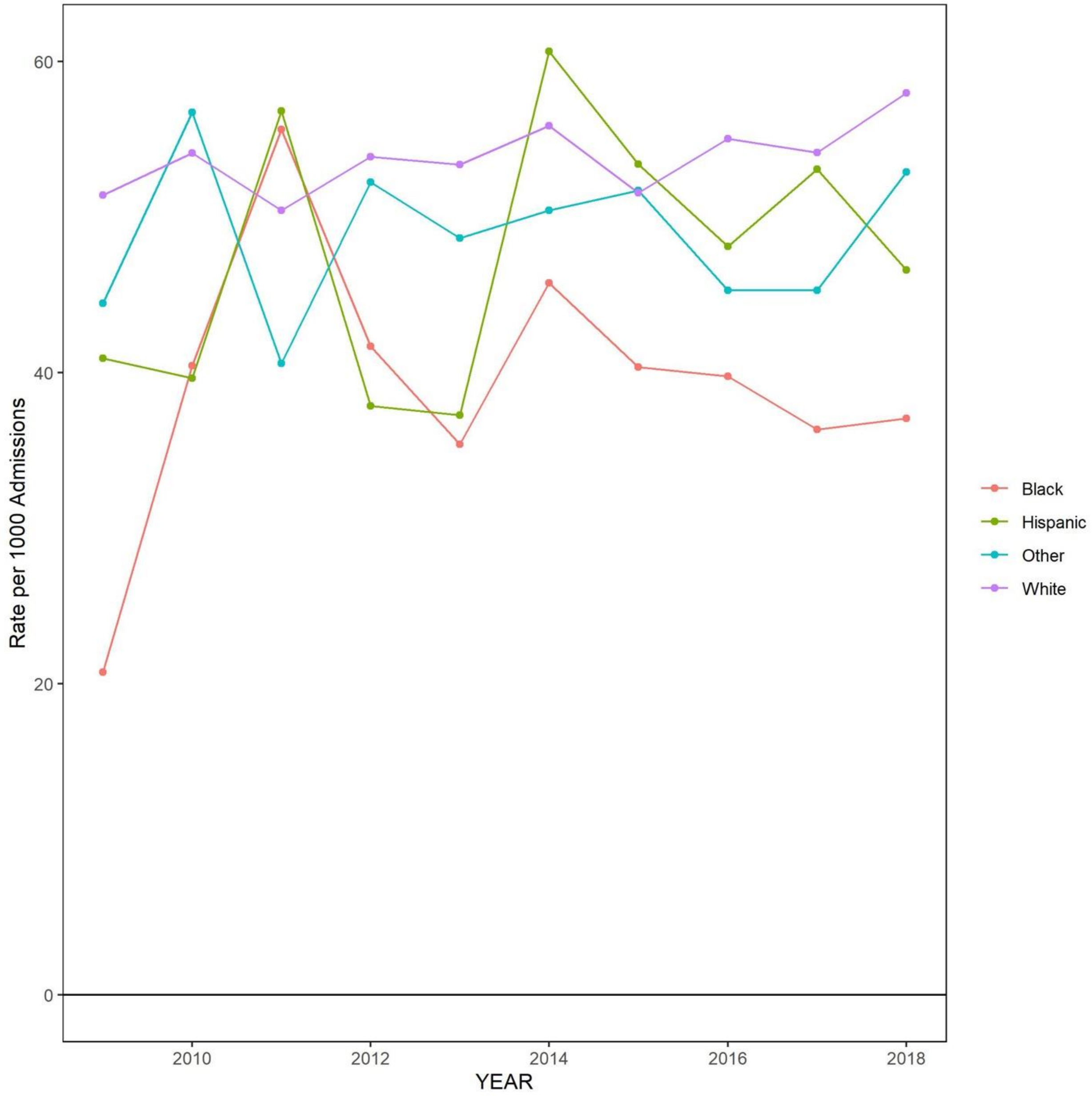

© 2023 Nephew LD et al. *JAMA Network Open.*

C. TIPS for ascites

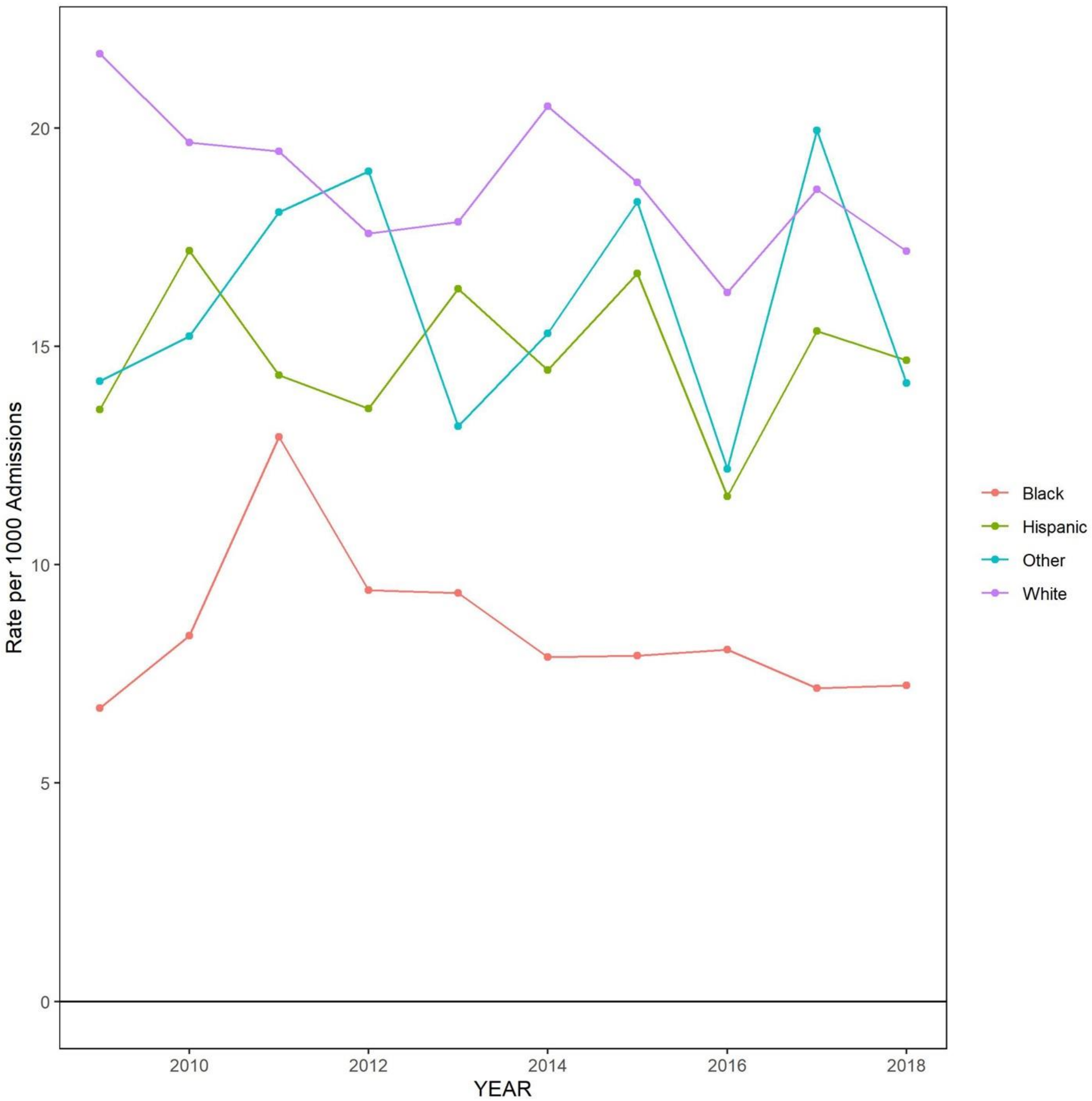

© 2023 Nephew LD et al. *JAMA Network Open.*

D. HD for AKI

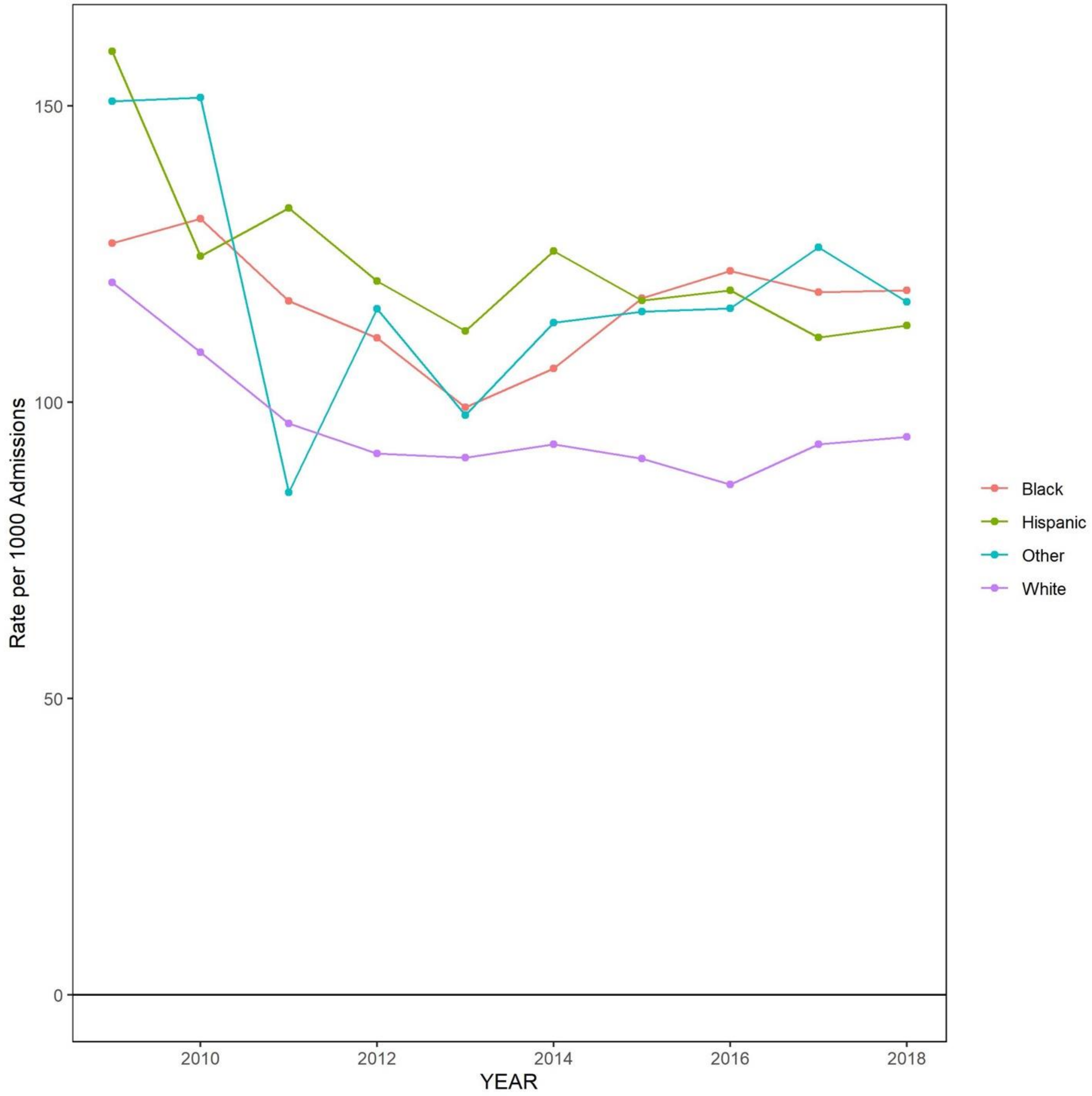

© 2023 Nephew LD et al. *JAMA Network Open.*

E. LT

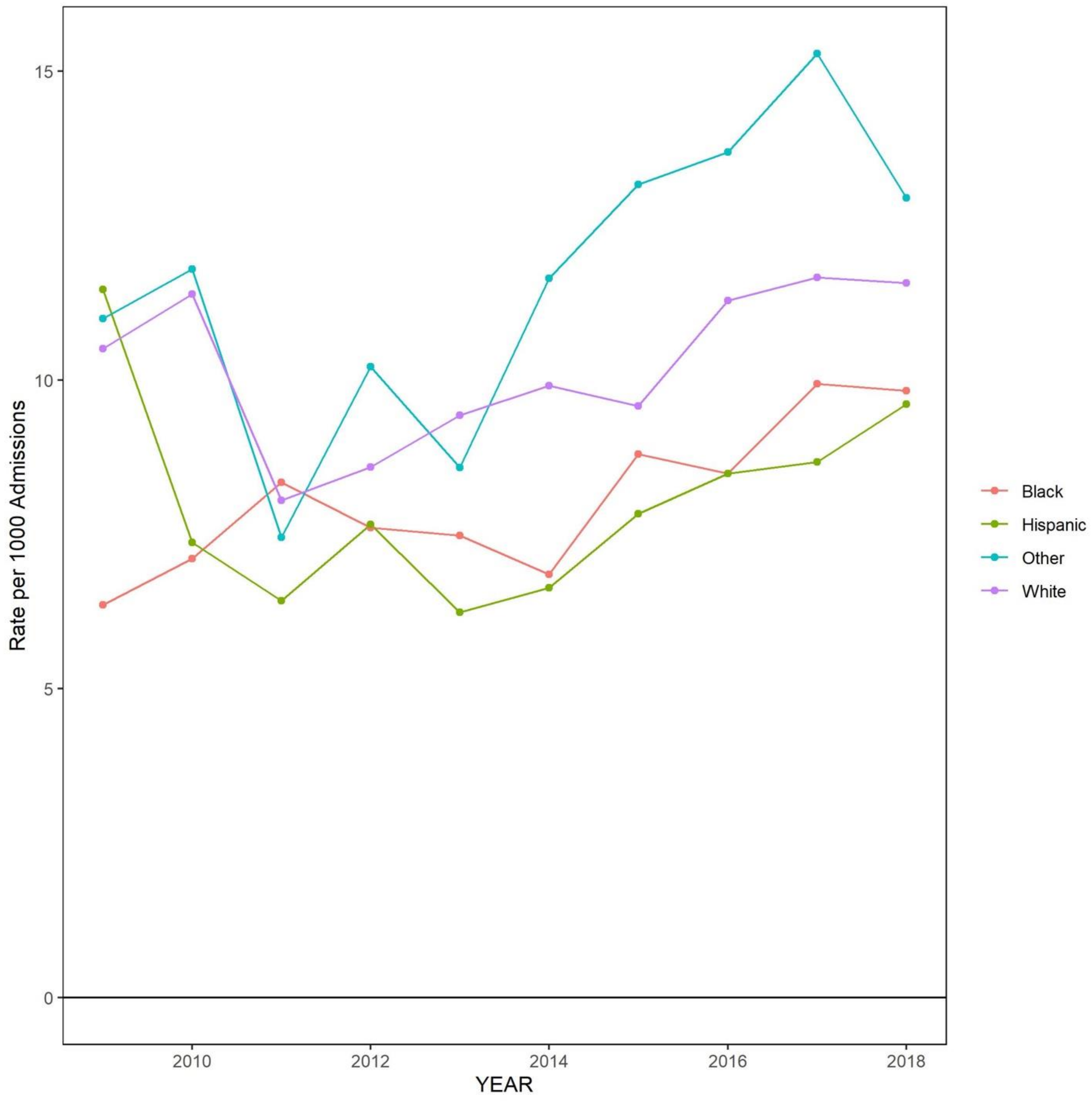

© 2023 Nephew LD et al. *JAMA Network Open.*

F. Mortality

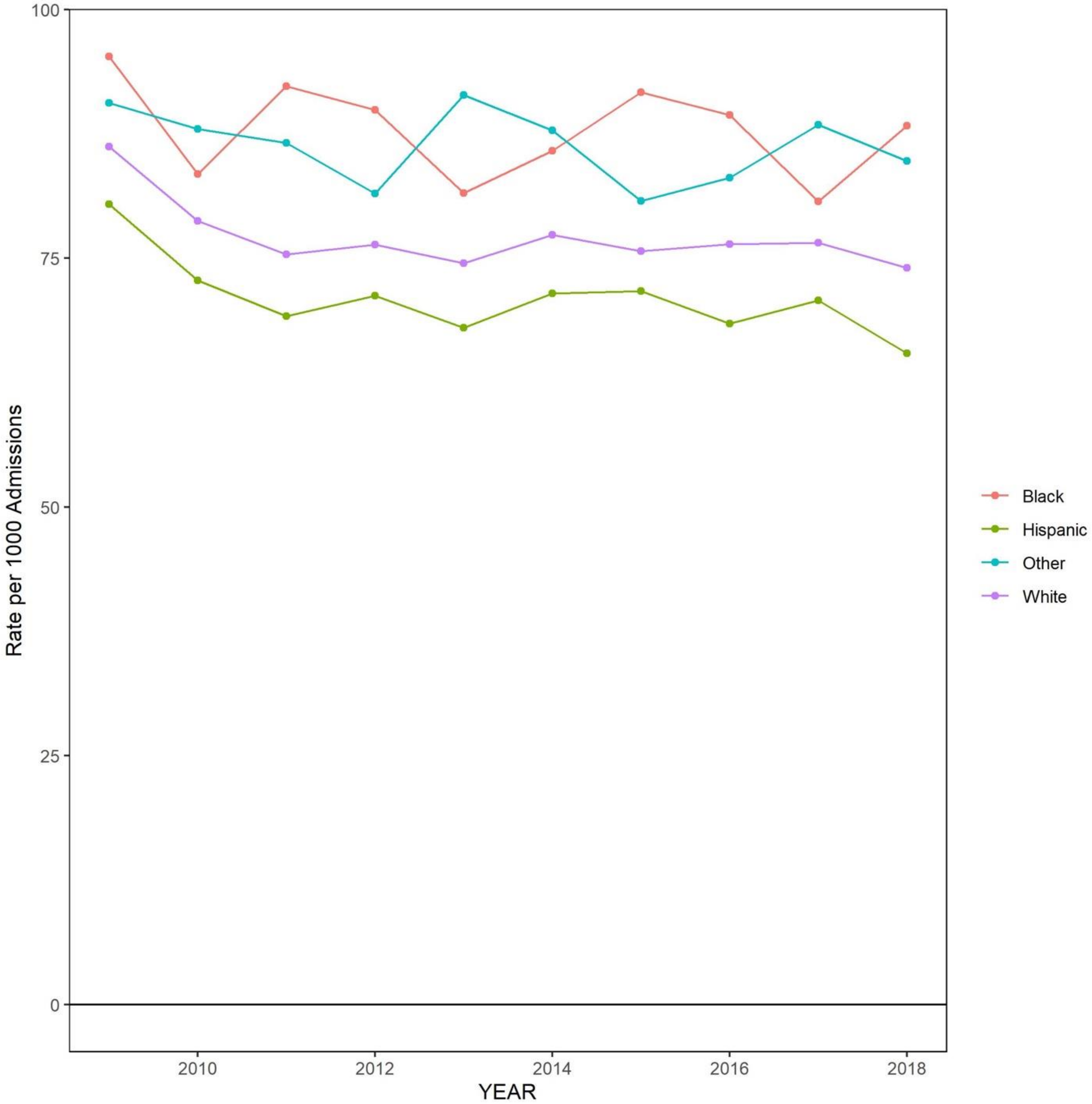

© 2023 Nephew LD et al. *JAMA Network Open.*
