# Supplementary material for: Trends in Racial and Ethnic Disparities in the Receipt of Lifesaving Procedures for Hospitalized Patients With Decompensated Cirrhosis in the US, 2009-2018
Source: JAMA Netw Open. 2023 Jul 20;6(7):e2324539. doi: 10.1001/jamanetworkopen.2023.24539 (PMC10359964; doi:10.1001/jamanetworkopen.2023.24539)
Supplement: Supplement 2. — Data Sharing Statement [file jamanetwopen-e2324539-s002.pdf]

Data Sharing Statement

Nephew. Trends in Racial and Ethnic Disparities in the Receipt of Lifesaving Procedures for Hospitalized Patients With Decompensated Cirrhosis in the US, 2009-2018. *JAMA Netw Open*. Published July 20, 2023. doi:10.1001/jamanetworkopen.2023.24539

Data
Data available: No

Additional Information
Explanation for why data not available: The analytic methods used in this study are detailed in the methods. The data will not be made publicly available by the research team but are available from the from Healthcare Cost and Utilization Project.
